# Colles’s Fracture

**Published:** 1896-07

**Authors:** J. B. Morgan

**Affiliations:** Augusta, Ga.


### COLLES'S FRACTURE.

BY J. B. MORGAN, M. D., AUGUSTA, GA.

The diagnosis and treatment of fractures generally are of as much, or more interest to the general practitioner as they are to the surgeon. By far the greater number of all fractures are treated by the doctors in general practice. The most com mon and frequent one with which we have to do is that of the lower end of the radius, commonly known as Colles's fracture, its frequency making it of special interest and of great practical importance. The cause is nearly always the same—catching on the hand in the act of falling. In falling, the open hand is thrown out, and the force of the fall is received first upon the palm. This results in hyperextension or forced dorsal flexion, which is checked by the strong mass of ligaments on the anterior side of the wrist-joint. As this extension is continued by the greater force on the hand, the ligament does not lacerate readily, but through its influence upon the lower end of the radius, this bone begins to yield on its anterior aspect, and as the force is increased, the line of fracture travels upwards and backwards until the fracture is complete. A momentary continuation of the same force will produce the usual displacement, causing the lower fragment to ride backward upon the upper, and frequently causing impaction of the firm posterior rim of the upper into the spongy substance of the lower fragment. From this explanation we would have no hesitancy in calling this a pronounced fracture by traction, and this view is generally accepted; but of late this theory has been disputed and the claim advanced that, during dorsal flexion of the hand, the upper row of carpal bones is forced against the dorsal prominence of the lower end of the radius, so that the fracture would be due to inflection rather than traction. It is immaterial to our purpose whether the fracture be the result of traction or of inflection.

Why the lower end of the ulna, as a rule, does not suffer injury, is easily understood from the anatomical arrangement of the parts, it having no direct connection with the wrist-joint itself. This fracture of the radius usually occurs about a half to one inch above the articular surface, and is generally transverse. The line of fracture varies within certain limits, but the manner of reposition is practically the same. The diagnosis of Colles's fracture is not difficult. The history of the accident, the characteristic deformity, and pain at the seat of the lesion, point you at once to the character of the fracture. A great deal has been said and written about the diagnosis of this typical fracture; but two points only are necessary to observe, in order to arrive at a correct diagnosis. The marked displacement of the whole hand toward the radial side of the wrist, and the relative position of the styloid processes of the ulna and radius. In the natural condition of the parts with the arm hanging by the side, the styloid process of the radius is on a lower level than that of the ulna; that is to say, nearer the ground. After fracture, this process is on the same or higher level than that of the ulna. The prognosis of the fracture depends in the main upon the treatment. Colles's fracture always produces deformity—the degree of deformity turns upon the amount of displacement of the broken fragments. A misinterpretation or diagnosis, or if rightly diagnosticated, neglected or improper treatment, will leave permanent this deformity. The first requirement in the treatment is to effect exact reposition. And I wish to emphasize most strongly, that this cannot be done by traction. Those of us who were taught, and have faithfully practiced this method, know how impossible it is. The vast number of permanent deformities from this injury is an unanswerable argument against its use. We can readily understand why traction is inefficient, when we consider for a moment the anatomical arrangements of the parts. In the first place, there is no muscle attached to the hand anywhere, that is attached to the radius below the line of fracture; therefore, in making traction at the hand, there are no muscular attachments by which the broken lower end of the radius can be pulled down into position. This being the case, the only thing that traction could accomplish, by the intervention of muscular attachment, would be to pull the upper and longer fragment of the radius into further displacement or firmer impaction, but the main reason why traction can not effect reposition of the fractured fragments, is on account of the action of the anterior and posterior annular ligaments during extension. These ligaments are continuous with, and may be regarded as thickened portions of the deep fascia of the forearm. They are strong, fibrous bands, arching over the front and back of the wrist-joint, firmly attached to the carpal bones below, and continuous with the deep fascia of the forearm above. The flexor and extensor tendons of the hand pass through and under these ligaments, in close contact with the lower end of the radius, this part of the bone being grooved for their reception. The effect of traction, therefore, would be to lengthen the longitudinal fibres of the deep fascia and annular ligaments at the expense of the transverse fibres. This would cause the ligaments around the wrist-joint to more closely bind the broken ends of the bone, and, more than this, the tendons which pass through and under the annular ligaments are also drawn strongly against the fractured bone, so that we would have a closely applied splint of tendons, held firmly in place by strong, flexible ligaments—making replacement by traction impossible. For the greater the extension the more closely will the tendons and ligaments hug the bone, and the more firmly the fragments become fixed in their displaced position. But while traction will not accomplish it, every case of Colles's fracture can be readily reduced by strong, forced dorsal flexion. This is best done under an anæsthetic, to avoid pain and produce complete relaxation of the tendons and ligaments about the wrist. It may require several attempts before exact reposition can b obtained, but with a little patience and care it can always be done. The best way to do this is with the patient's hand in pronation, you grasp his forearm with one hand, in such a way that while the radius is firmly held, your thumb rests just above the line of fracture. With the other hand, you grasp the hand of the patient, so that your thumb presses firmly upon the back of the lower fragment. The hand is now carried strongly back towards the dorsal aspect of the radius in forced and extreme dorsal flexion, until you feel by palpation that the lower fragment has become unlocked, and can be pushed into place by your thumbs, while at the same time, the patient's hand, under strong extension, is carried into the normal position. If this manœuvre is properly done, and the fragments accurately replaced, almost any kind of a dressing which keeps the fragments at rest, will give a good result. The peculiar character of of the displacement in this fracture and the constant difficulty experienced in obviating deformity, have greatly stimulated inventive ingenuity, and resulted in a number of specially devised splints. We have the Gordon splint, the Coover splint, the Levis splint, the Hamilton splint, the Nelaton splint, the Bond splint, the Hays splint, and a great number of others. I have neither the time nor inclination to discuss the merits and demerits of these various splints. All of them are more or less faulty; none of them perfect. Most of these splints were devised upon the assumption that, by bearing the hand strongly to the ulna side, the fragments of the radius are brought more exactly into apposition, and more easily and effectually retained—a supposition that takes for granted two things; first, that there exists an overlapping of the fragments, either through the whole extent of their broken surfaces or especially towards the radial side, or, that the upper end of the lower fragment is inclined to fall against the ulna, or that all of these several conditions coexist; and, second ly, that if such displacements do exist they can be remedied by fixing the hand in strong ulna flexion. The answer to the first supposition is, that Robert Smith and others, who have made a particularly careful examination of all the specimens they could find, in the various pathological collections, tell us that none of these displacements have been found to exist; but that the displacement is backwards and somewhat upwards; so that in bones in which unreduced fractures of this kind have consolidated, the posterior surface is found to be considerably shortened, while the anterior retains its normal length, and, secondly, in order that this position may prove effective, there must be some point upon which to act as a fulcrum. It is of no use that we rotate the hand for the purpose of making extension unless there can be found a resistance or fulcrum upon which the rotary motion may be performed. It is not in the ulna certainly, for that bone has no point of contact with the carpal bone. When the lower end of the radius is broken and the ligaments of the joint more or less torn, the ulna, although thrust downwards much farther perhaps, than it could ever descend in its normal state, still fails to find a support, and spreading wider and wider from the radius as it is thrust farther upon the hand, no limit can be given to its progress, except the capacity of the joint to admit of motion in this direction; and, further, this position is unnatural and exceedingly painful. In the normal hand this position when forced, can not be endured beyond a few seconds and the pain must be infinitely worse, in this position, when the ligaments are torn, the bone broken and the wrist inflamed and tender; and if persisted in, will have the effect of greatly increasing the inflammation and leaving, in nearly every instance, an unsightly, crooked and permanently stiff wrist, to the great inconvenience of the patient and annoyance of the doctor. It only remains for us to determine the form of splint which ought to be preferred,and to describe its mode of application. The best temporary and, in most cases, the best permanent dressing is

Wyeth's modification of Pilcher's. It is simple and highly satisfactory, and is applied as follows: Roll two pieces of a bandage, two inches and a half wide, into a compress about as thick as the little finger. After reduction is complete and the hand brought back into the straight position, place one compress along the inner aspect of the ulna, extending from the anterior margin of the carpus upward, the other exactly parallel with this, along the outer border of the radius, over the styloid process. While these are held firmly in position, secure them by strips of adhesive plaster, one inch in width, wound securely around the wrist and arm, from the upper end of the compresses to the lower end. The hand and arm should be carried in a sling, and the dressing worn for two or three weeks. This dressing is neat, and while holding the fragments accurately and firmly in apposition, has the additional advantage of allowing the freest possible mobility of the wrist and fingers, thereby greatly lessening the probability of temporary or permanent stiffness.

The plaster of paris dressing is a most excellent one, and is to be preferred in old people where there is firm impaction which you do not care to break up, or in cases where the fragments are more or less comminuted. It should be applied, from the lower border of the metacarpus to the middle third of the forearm—with the patient's hand in the straight position. A straight dorsal splint may also be employed, but it is not as desirable as the plaster of paris. Under no circumstances use the angular, crooked or pistol shaped splint, and no form of splint should be allowed to project beyond the metacarpus. The fingers must remain freely moveable, to prevent stiffness of the joint. Motion should be encouraged; at first, limited and careful, later, active and free. In aged patients, where you have more or less firm impaction of the broken ends, reduction should not be attempted, as it is better to have a crooked, deformed wrist, than a failure in bony union, and impaction favors the consolidation of the fractured bone.

